# Apps for oral hygiene in children 4 to 7 years: Fun and effectiveness

**DOI:** 10.4317/jced.55686

**Published:** 2019-09-01

**Authors:** Francesca Zotti, Angelo Pietrobelli, Luciano Malchiodi, Pier-Francesco Nocini, Massimo Albanese

**Affiliations:** 1 Researcher, Section of Dentistry and Maxillofacial Surgery, Department of Surgical Sciences, Paediatrics and Gynecology, University of Verona. Policlinico G. B. Rossi. Piazzale L. Scuro n.10, 37134. Verona, Italy; 2 Associate Professor, Section of Paediatrics, Department of Surgical Sciences, Paediatrics and Gynecology, University of Verona. Policlinico G. B. Rossi. Piazzale L. Scuro n.10, 37134. Verona, Italy & Pennington Biomedical Research Center, Baton Rouge, 70808, LA, (USA); 3 Associate Professor, Section of Dentistry and Maxillofacial Surgery, Department of Surgical Sciences, Paediatrics and Gynecology, University of Verona. Policlinico G. B. Rossi. Piazzale L. Scuro n.10, 37134. Verona, Italy; 4 Chair, Professor, Section of Dentistry and Maxillofacial Surgery, Department of Surgical Sciences, Paediatrics and Gynecology, University of Verona. Policlinico G. B. Rossi. Piazzale L. Scuro n.10, 37134. Verona, Italy; 5 Associate Professor, Section of Dentistry and Maxillofacial Surgery, Department of Surgical Sciences, Paediatrics and Gynecology, University of Verona. Policlinico G. B. Rossi. Piazzale L. Scuro n.10, 37134. Verona, Italy

## Abstract

**Background:**

Nowadays apps in preschool age are largely used in learning improvement. The aim of this work was to test effectiveness of apps in improving oral hygiene in children patients aged from 4 to 7 years and evaluating correlation between parents educational attainment and children oral hygiene.

**Material and Methods:**

100 patients aged from 4 to 7 years were randomly assigned by an external office in the study group (SG: 32 females, 18 males) and in the control group (CG: 28 females and 22 males). Plaque index (PI) and carious lesions localisation were detected. At baseline all patients and one of the parents were instructed at chair-side about the proper oral hygiene techniques. SG patients were also given app as an aid in oral hygiene practice. Follow-up was 12 months. Measurements were made every three months at chair-side visits. Information about children compliance in oral hygiene and educational level of parents were obtained by questionnaires at t0 and after 12 months.

**Results:**

SG patients showed stronger oral hygiene and PI lower than those in CG. Questionnaire showed higher compliance of SG patients and parents educational level seemed to affect children oral hygiene.

**Conclusions:**

Apps in children allowed achieving encouraging results with improvement of oral hygiene and health.

** Key words:**Apps, oral hygiene compliance, children oral hygiene, motivation, educational attainment.

## Introduction

Dental caries are one of the most widespread disease and they are called Early Childhood Caries (ECC) when affect subjects younger than 71 months. Prevalence of ECC in Italy is 14,4% whereas of Severe Childhood Caries (S-ECC) is 5,9% (1) . However significant variations are detectable depending on different region of country (2).

Carious disease has a multifactorial aetiology, mainly related to the eating habits and lifestyle (3). When tooth decay affects children, it is often necessary to take into account the motivation of these patients to maintain proper hygiene constantly and their degree of manual skills (1). The preschool period is a crucial time when eating habits and lifestyle are radically established, children stop being fed by mothers and begin to show their own tastes. Children manual skills are very poor as well as the motivation to oral hygiene and often parents are forced to take part in the oral hygiene practices (2) . Even now, there are children younger than 2 years who are aware of oral hygiene techniques and who do not visit a dentist until mixed dentition with consequent impact on their oral health and expected ECC (3). The level of education and socio-economic status of parents often influences oral hygiene of children, as well as motivation for periodical dental visits oral health care overall (4–7). At present days, however, technology is largely used in improving motivation and compliance in health management and in oral health (8) and it might be a point to take into account also for improvement of children engagement and compliance maintenance.

Children are accustomed to using technology enabling its use in their education. Several apps and tools are specifically developed for training and teaching children with subsequent increasing distribution (9) and often avoiding need to have proper training to approach them. In dentistry, technologies and today apps to educate and motivate in oral health maintenance are broadly available (8).

Aim of our study was to test the effectiveness of some media on maintaining and improving oral hygiene in young patients aged from 4 to 7 years in terms of plaque index and dental caries incidence in one year of observation. A secondary aim was to evaluate potential correlation between educational attainment of parents and children oral hygiene maintenance and improvement.

Null hypotheses were that the use of technological aids in children motivation was not significant and that there were no substantial differences between those who use media and who does not use them in the maintenance of proper oral hygiene.

## Material and Methods

One hundred patients aged from 4 to 7 years related to three private dental practices were recruited and randomized in two groups using a software by an external office.

- Criteria to be enrolled were:

No food allergy and / or environmental

No restrictions of diet(10)

Negative general medical history 

No medication

No interceptive orthodontic treatment present or in history

No previous trauma

No early loss of deciduous teeth

Speaking Italian language

**Figure 1:**
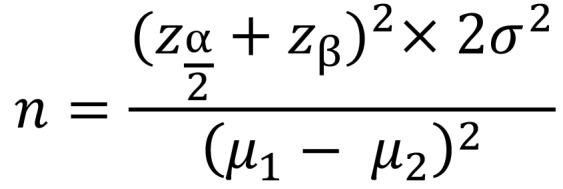
Formula.

An a priori sample size (n) calculation, with the plaque index (PI) as the main outcome, was performed, fixing a power (ß) of 90% (zß= 1.28) and an α of 5% (zß/2= 1.96), considering clinically significant a difference of 0.25 in the means (µ) of the outcomes between the two groups, with a standard deviation (σ) of 0.5 estimated on present literature, using the following formula (9): (Fig. 1).

In order to prevent inhomogeneity among the groups, the sample was stratified according to the following parameters:

Breakdown by age: 2 groups. In the first group subjects from 4 years to 5 years and 6 months. In the second group subjects aged from 5 years and 6 months to 7 years.

Breakdown by gender

Breakdown by the Decay, Missed, Filled Teeth Index (Dmft Index): Dmft up to 1.9 and greater than 1.9

Stratification allowed homogeneity of groups for gender, age and oral health conditions at baseline, and thereafter patients were randomly allocated in two different groups by an external office.

 Table 1 shows sample distribution after randomization and allocation ( Table 1).

**Table 1:**
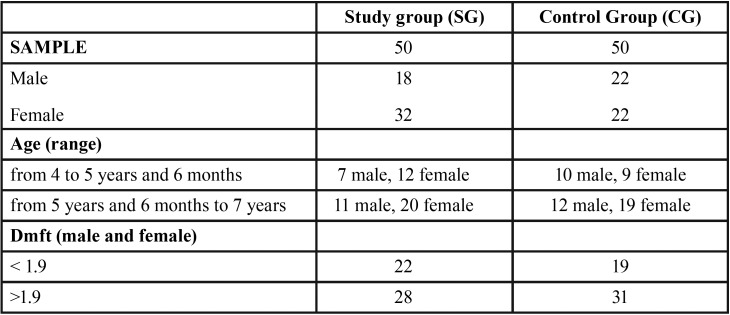
Sample distribution at baseline.

Parents were asked domiciliary oral hygiene procedures were carried out by children exclusively. Written informed consent was requested and obtained from both the parents, as well the consent to use apps for smartphone.

At baseline (t0) a dental examination to determine degree of oral hygiene and the presence or absence of carious lesions was carried out(10,11).

Following outcomes were collected during the visit:

Plaque Index (PI according Silness & Löe)(12) 

Presence or absence of carious lesions

Localization of carious lesions: pits or fissures, interproximal, occlusal surfaces teeth.

**Figure 2:**
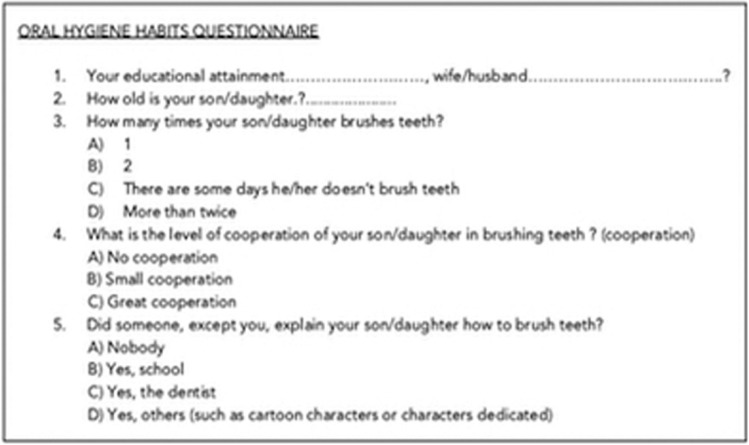
Oral hygiene habits, questionnaire answered by parents (both groups) at baseline.

In addition one of the parents answered a multiple choices questionnaire on children oral hygiene habits, level of parental educational attainment was also included in first questionnaire (Fig. 2).

After dental examination, all patients and their parents were instructed at chair-side by a dental hygienist, using a model, to correct domiciliary oral hygiene technique applied for all teeth and surfaces including only back-and-forth movements(13). Patients were then asked to reproduce the brushing technique on the model in the presence of parents.

Parents of SG patients also received instructions to download a specific app for children designed to increase compliance in oral hygiene. Two different apps were chosen depending on the age of patients: Time2Brush (developer Bunner Mobile, retailer Bunner Inc © 2012 GlaxoSmithKline) for patients over 5 years of age and Brusheez- The Little Monsters Toothbrush Timer (developer Shondicon LLC) for children up to 5 years, as indicated in the specifications of developers.

App format and purpose were common to both applications, consisting in a game in which a fictional character could serve as a motivator for oral hygiene and stopwatch (2 minutes) to more effectively carry out regular proceedings. In addition, both apps allowed, in response to the minutes of use, obtaining bonus to customize the main character of the game or even unlock new characters.

The recommendation for parents was to submit the apps to children and to use them as an aid in oral hygiene motivation twice a day.

Participants of CG were only instructed for oral hygiene and they did not take the app.

All patients were provided with toothpaste and a toothbrush that was replaced at each quarterly inspection (Elmex Children’s Toothbrush: 3 to 6 years of age and Junior Elmex Toothbrush: children older than 6 years; Elmex Children Toothpaste; Colgate-Palmolive S.p.a, NY, USA).

**Figure 3:**
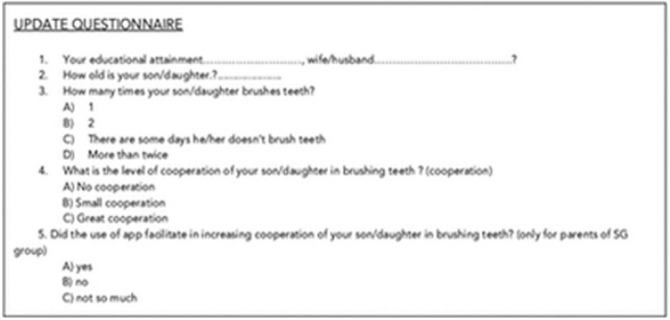
Update questionnaire answered by parents (both groups) at t4.

Regular visits were scheduled every three months (3 months: t1, 6months: t2, 9 months: t3, 12 months: t4) to assess oral hygiene status and outcomes parameters checked in the baseline visit (PI, presence / absence of carious lesions, localisation of carious lesions) in both groups. At the end of observation period (t4), a short questionnaire about the children habits in oral hygiene was administered to parents of all children as in Figure 3 (update questionnaire).

Each visit was carried out by the same dentist in order to evaluate study outcomes according with the following rules:

Measurement of plaque index (PI), according Silness J & Löe (12): as not all patients had the same permanent teeth, elements A, H, I, J, M, S were used as references. Each teeth surfaces (buccal, lingual, mesial and distal) were scored 0 to 3 for each tooth, the major value was considered, mean of values of all teeth was then calculated. PI scoring is considered as following: 0 no plaque, 1 slight deposit of plaque at gingival margin, 2 moderate deposit of plaque covering less than half of the surface, 3 Important deposit of plaque covering more than half of the surface .

Presence/absence of carious lesions and their location on tooth: teeth were cleaned with toothbrush professional prior to their observation and a jet of water and air was blown. Teeth were examined clockwise, from upper right quadrant to the lower right.

Degree of children cooperation in oral hygiene: evaluated by questionnaires at the beginning (t0) and at the end of the study (t4), for each group of patients and correlated to parents’ educational attainment. Answers evaluated were: Null cooperation, small cooperation and high cooperation. Parent’s educational levels analysed were: graduation, high school and junior high school.

Differences between groups of PI values at different time-points were assessed by unpaired two samples t-Test. Differences of PI within groups at different time-points were assessed by one-way ANOVA with Bonferroni correction. Degree of cooperation in oral hygiene and its correlation to educational attainment of parents was analysed by Chi-square test with Pearson’s R. Tests were considered significant for *P*≤ 0.05. Pearson’s R was interpreted as following: +1 indicated positive correlation, -1 negative correlation, 0 no correlation.

Descriptive analysis was carried out to better evaluate other outcomes (presence/absence of carious lesions, their location).

All statistical analyses were performed using Statistical Package for Social Sciences Version 22.0 (SPSS Inc., Chicago, IL).

## Results

**Figure 4:**
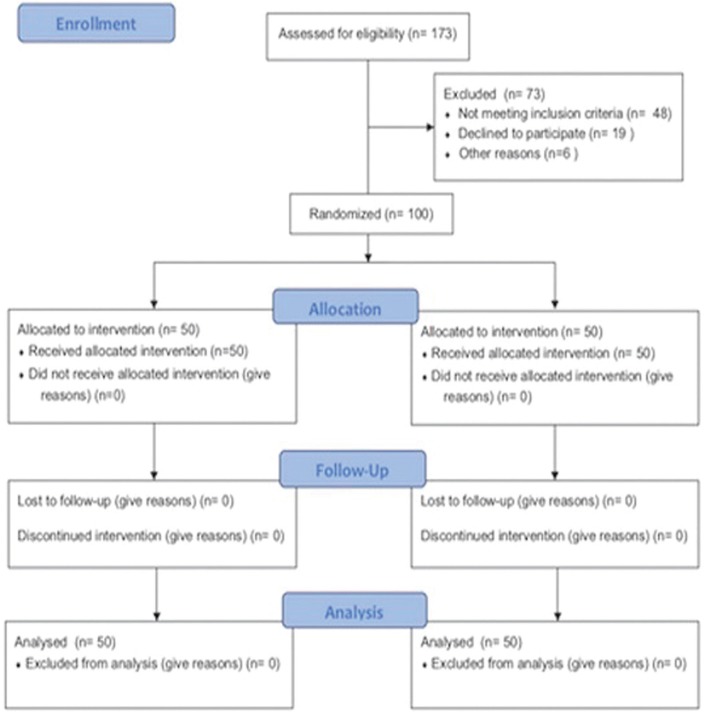
CONSORT flow diagram.

No drop-out was registered during the follow-up period in both groups (Fig. 4). Each outcome was individually analysed for each group of patients and shown in following tables. Values of PI are represented as mean ± standard deviation, P values of ANOVA test for differences within groups are shown in  Table 2. Differences in PI values between groups were found to be statistically significant (*P* ≤ 0.001) in t1, t2, t3, t4 whereas differences were not detected between groups at t0.

**Table 2:**
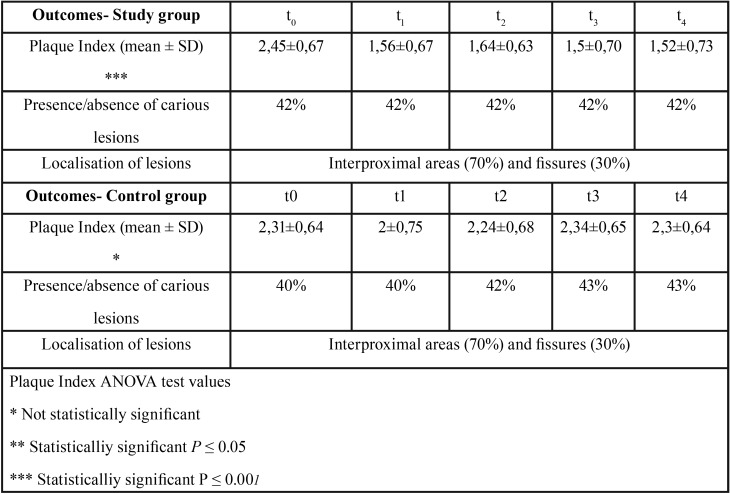
Within groups differences at different timepoints.

No cavities were detected in permanent teeth.

Correlation between parents’ educational attainment and oral hygiene maintenance of children in t0 and t4 was investigated by questionnaire and a positive correlation was found both at t0 both at t4. Children’s oral hygiene and maintenance seem to be affected by parents’ educational attainment as well the improvement of oral hygiene degree detected in SG.

## Discussion

Children are more at risk of dental decay onset because of lack of cooperation and difficulty in daily oral hygiene performing (14). Furthermore, evidences show that, even today, many children do not visit a dentist before of development of permanent teeth14 and it seems to be especially related to socio-economic level of families and to knowledge about the dental care needing in childhood (4–7,15). In order to better investigate these issues, literature suggests to administer questionnaires to parents and several studies have assessed caries risk by using surveys about oral hygiene habits and attendance at dental visits, as well as about parents carefulness in early detecting teeth diseases (16,17).

Cooperation of children is a challenging goal to reach, especially in oral hygiene maintenance, and this work aimed to test the effectiveness of new technologies and media supports in improving this issue. Furthermore, we aimed to correlate the educational attainment of parents and the degree of cooperation of children in oral hygiene 17, based on evidences of correlation between onset of tooth decay in children and socio-economic status of their family (6,7).

It is surely difficult to fix strong assessment methods to define and standardize oral hygiene parameters in children and scientific literature provides Dmft as most widely used index, however gum’s health indices or indices of presence of plaque exclusively used in children are not given. This is exactly why we used a modified Silness & Löe (12) index to estimate the presence of plaque and we clinically evaluated the presence and localisation of carious lesions; aimed to perform a rapid assessment, workable in dental offices without economic costs and additional time, avoiding trying children patience and cooperation.

Results of this study, compared to recent data in the literature, show an improvement of compliance in oral hygiene performances of children when they are engaged by technological supports, properly designed to enhance this issue, even though the low educational attainment of parents. This study shows that introduction of apps for domiciliary oral hygiene is almost evenly well received in all young patients (18) .

Use of technological devices and applications in oral hygiene management seemed to be effective in improving compliance of young patients and recent literature provided encouraging results in this field, especially in orthodontic patients (9) and this work was based on these considerations. Media tools were found to be attractive for young patients undergoing long-term dental therapies, as orthodontic treatments are, and results in terms of compliance were encouraging; we assumed that maintenance and improvement of domiciliary oral hygiene in children had the same critical points to bypass. Children’s compliance, in fact, is difficult to kept constant overtime and it shall be indicated to improve oral hygiene conditions in childhood, given that prevention of dental caries is of crucial importance and that children are lacking in this field (14). In this regard, a key point to take into consideration is the availability of technological tools used to enhance and maintain compliance over time, avoiding lack of interest of young patients. Technological development comes to the aid supplying new properly designs to deliver regular updates and to challenge users with continuous contests and goals to reach: aspect of great importance when young patients are involved (19,20). Parents’ role are essential in this regard providing important feedback about cooperation and engagement of children; it could become a further resource to better customize the choice of media supports used to increase compliance. This concept becomes of even more practicability especially in private practice, where dentist, young patients and their parents have a closer and more continuous relationship compared with those of great hospital facilities (14,21,22).

Analysis of the results shows that oral hygiene conditions at baseline were similar in two groups, only slightly greater in the control group but not statistically different. Degree of oral conditions in CG did not change consistently during the observation period and differences between PI values into CG were not statistically significant. This might be interpreted in terms as an indication that chair-side instructions did not reach the goal of improving compliance, but they contributed in keeping constant levels of oral hygiene over time.

Input of download apps for oral hygiene, on the other hand, allowed obtaining better results in terms of PI, thus proving that technologies could be a good support in increasing cooperation of children.

A decrease in the PI, nevertheless not statistically significant, in the first three months of observation was also detected in CG (PI in t0=2,3 vs PI in t1 = 2), probably due to training received by children at the beginning of the study and the new element introduced in their routinely oral hygiene habits. However PI reverted to values collected at baseline after three months of follow-up.

In SG a decrease of PI was observed in t1 and its values remained constant over time; in this regard it could be argued that different entity of decrease of PI in two groups in the first three months might be due to use of apps support by SG patients. In fact, decrease of PI in SG was observed from 2,45 to 1,56, whereas in the CG the extent of decrease was lower: from 2,3 to 2. It might be possible that the apps downloaded by SG patients encouraged a greater care in performing oral hygiene procedures.

In this work we adopted outcomes easy to be detected and analysed in order to be as close as possible to daily dental practice conditions, where chair-time is often short, and to little patience of children. We believe that is crucial, when young patients are approached in dental practice, to be as effective as possible in order to not try their compliance and to reach treatment or prevention goals.

We limited our investigation of carious lesions to those lesions detectable by probing and identifiable to the naked eye. This choice, often in disagreement with literature that differentiates enamel and dentine caries for a restorative approach, depended on our need to merely study the influence of motivation in development of dental caries whereas methods of dental caries assessment were not under study (23). Furthermore, it is difficult to assess quarterly caries development because visible changes in teeth’s surfaces are limited in a short period of time, however the trend of carious disease seems to be related to PI values and variations of this index were still observed in the two groups at each check visit (24). These data are in agreement with the literature where the presence of plaque is correlated with the occurrence of caries (25). For these reasons it could be indicated to read results of presence of caries in the long term (t4) and to consider values of PI most relevant at each time-points (t1, t2, t3, t4). The localisation of lesions was not different in the two groups. According to literature, interproximal lesions were found in a percentage higher than those of the occlusal surfaces in both groups, arguably due to greater difficulty of cleaning interproximal surfaces compared to the occlusal surfaces (26).

We did not evaluated objectively the degree of fun of using apps for domiciliary dental hygiene. We think this outcome might be worth a special and separate study. However we should assume that the apps were considered funny by children of SG because their compliance in maintenance of dental hygiene was higher than compliance registered in CG. Parents reported also a cooperation of children of SG and this aspect would tend to suggest a good approval rating.

To deeply investigate degree of fun in using apps was not subject of this study. Of course it could be of great interest to give all patients (or parents) who used apps a dedicated questionnaire to investigate the fun and the niceness of different apps. In this field, in further studies, it might be appealing to compare different apps in order to discover the most appreciated by children of various ages.

Concerning degree of cooperation and parents’ educational attainment, our results showed high correlation between educational level of parents and cooperation of children both in t0 and in t4, demonstrating that cooperation might be particularly influenced by this feature. These findings are in accordance with those in literature (4–7), even if we investigated merely educational level, whereas cited works deeply analysed more aspects of socio-economic status of parents related to oral hygiene maintenance of children. However, educational attainment of parents was found to not affect substantially the cooperation level of children in t4, in fact it evenly increased in SG in all patients. It is surely encouraging, proving that technologies and multimedia apps could provide an improvement in compliance of children without prejudice to parents’ educational level. Differently, in patients of CG, degree of cooperation in t4 showed a general minor improvement and cooperation was found to slightly rise, probably explained by the fact that in this group apps have not been used.
